# Acute Toxicity and Gastroprotective Effect of the Schiff Base Ligand 1*H*-Indole-3-ethylene-5-nitrosalicylaldimine and Its Nickel (II) Complex on Ethanol Induced Gastric Lesions in Rats

**DOI:** 10.3390/molecules171012449

**Published:** 2012-10-22

**Authors:** Mohamed Mustafa Ibrahim, Hapipah Mohd Ali, Mahmood Ameen Abdullah, Pouya Hassandarvish

**Affiliations:** 1 Department of Chemistry, University of Hail, Hail 81451 , Saudi Arabia; 2 Department of Chemistry, University of Malaya, Kuala Lumpur 50603, Malaysia; Email: hapipah@um.edu.my; 3 Department of Molecular Medicine, University of Malaya, Kuala Lumpur 50603, Malaysia; Email: mahmood955@yahoo.com (M.A.); pouya3132@gmail.com (P.H.)

**Keywords:** Schiff bases, metal complexes, tryptamine, anti-ulcer, cimetidine, histology

## Abstract

The present study was performed to evaluate the gastroprotective activity of Schiff base ligand derived from the condensation reaction of tryptamine (an indole derivative) and 5-nitrosalicylaldehyde (TNS) and its nickel (II) complex against ethanol-induced gastric ulcer in rats. The compounds were orally administered with low (30 mg/kg) and high (60 mg/kg) doses to ulcer-induced Sprague-Dawley rats. Macroscopically, the ulcer control group exhibited severe mucosal injury, whereas pre-treatment with either cimetidine or TNS and its nickel (II) complex each resulted in significant protection against gastric mucosal injury. Flattening of gastric mucosal folds was also observed in rats pretreated with TNS and its nickel complex. Histological studies of the gastric wall of ulcer control group revealed severe damage of gastric mucosa, along with edema and leucocytes infiltration of the submucosal layer compared to rats pre-treated with either cimetidine or TNS and its nickel (II) compound, where there was marked gastric protection along with reduction of edema and leucocytes infiltration of the submucosal layer. Acute toxicity study done on mice with a higher dose of 5 g/kg of TNS and its nickel (II) complex did not manifest any toxicological signs. Research finding suggest that TNS and its nickel (II) complex could be considered as effective gastroprotective compounds.

## Abbreviations

ICR: Institute of cancer research
S.E.M: Standard error mean

## 1. Introduction

Schiff bases which are a class of compounds containing an azomethine group (-C=N-), have drawn attention for a long time due to their biological activities [1]. Indole and its derivatives are secondary metabolites that are present in most plants such as unripe bananas, broccoli, clove, almost all flower oils (e.g., jasmine and orange blossoms) and coal tar [2,3]. In the pharmaceutical field it has been discovered that it acts as an antimicrobial and anti-inflammatory [4]. A great deal of information regarding the properties of synthetic Schiff bases of potential biological interest has come to light during the last few years, as several of these compounds were characterized and used as models for a series of systems [5,6,7,8,9]. Metal complexes have been used widely to treat cancer, arthritis and diabetes [10]. Copper (II) complexes are known to be effective against rheumatoid arthritis and they also show anti-ulcer activity [11,12]. This is significant because gastrointestinal irritation often precludes treatment by other antiarthritic drugs. This is in line with the role of copper in generally preventing gastrointestinal damage by acidic anti-inflammatory agents [12]. 

In a previous paper we reported the anti-ulcerogenic activity of the Schiff base derived from tryptamine and 5-chlorosalicyldehyde and its nickel and copper complexes [13]. Continuing with this field of study we highlight here the anti-ulcer activity of a nitro-substituted Schiff base analogue and its nickel (II) complex.

## 2. Results and Discussion

### 2.1. Chemistry

**Scheme 1:**
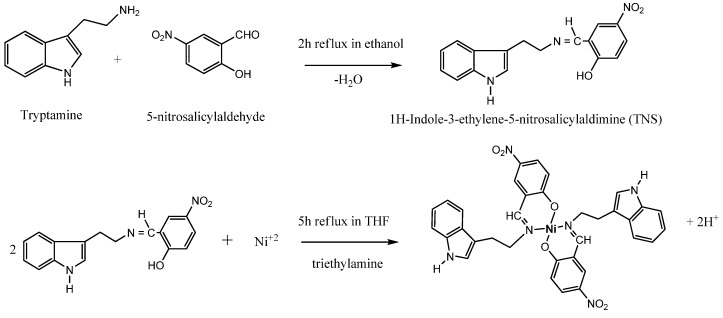
Reaction routes.

The reaction of tryptamine with 5-nitrosalicylaldehyde afforded the ligand 1*H*-indole-3-ethylene-5-nitrosalicylaldimine (TNS) in a good yield. The reaction of this ligand with nickel (II) salts gives the corresponding nickel (II) complex with 1:2 metal-to-ligand ratio, as shown in Scheme 1 below.

### 2.2. Results

#### 2.2.1. Acute Toxicity Study

No mortality occurred amongst the mice with dose levels of 2 g/kg and 5 g/kg of the Schiff base TNS and its nickel (II) complex during the study period. Behavioral observations did not show evidence indicative of significant drug toxicity. No abnormal macroscopic and microscopic changes of the organs were observed following macroscopic necropsy and histopathological examinations. The results suggested that the oral LD_50_ of these compounds was greater than 5 g/kg.

#### 2.2.2. Macroscopic Evaluation of Gastric Lesions

**Figure 1:**
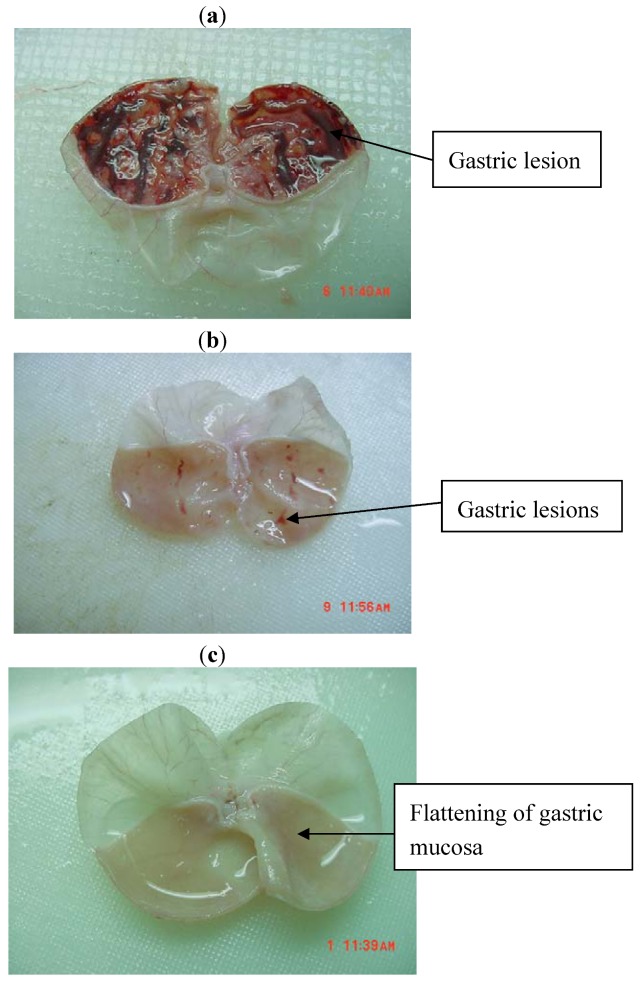
(**a**) Macroscopic appearance of the gastric mucosa in a rat pre-treated with 10 ml/kg of 10% Tween-20 (ulcer control). Severe hemorrhagic mucosal lesions are seen in the gastric mucosa. (**b**) Macroscopic appearance of the gastric mucosa in a rat pre-treated with Cimetidine (50 mg/kg). Injuries to the gastric mucosa are milder compared to the injuries seen in the ulcer control rat. (**c**) Macroscopic appearance of the gastric mucosa in a rat pre-treated with TNS (60 mg/kg). No injuries to the gastric mucosa are seen, and showed flattening of gastric mucosa.

Rats pre-treated with either 50 mg/kg cimetidine or the Schiff base TNS and its nickel (II) complex before being given absolute alcohol had significantly reduced areas of gastric ulcer formation compared to rats pre-treated with only 10% Tween-20 [Table 1, Figure 1a–c]. Moreover, the compounds significantly suppressed the formation of the ulcers and it was interesting to note the flattening of gastric mucosal folds in rats pretreated with these compounds. It was also observed that protection of gastric mucosa was more prominent in rats pre-treated with both high and low doses of TNS ligand and high dose of the nickel (II) complex (Table 1). The significant inhibition of gastric ulcer in pretreatment with the compounds was compared with cimetidine which is a standard drug used for curing gastric ulcer.

**Table 1:** Effect of TNS and its nickel (II) complex on ulcer area, mucus weight, pH and inhibition percentage in rats.

Compound	Total ulcer area (mm^2^)	Mucus weight (g)	pH	% Inhibition
	(Mean ± S.E.M)			
Tween-20	1438 ± 58 ^a^	0.58 ± 0.05	2.00 ± 0.01	-
(ulcer control)				
Cimetidine	168 ± 5 ^b^	0.61 ± 0.05	3.00 ± 0.01	88
(+ve control)				
TNS (HD)	3.6 ± 0.2 ^c^	1.33 ± 0.05	4.22 ± 0.01	100
TNS (LD)	3.6 ± 0.2 ^c^	1.60 ± 0.05	4.11 ± 0.01	100
(TNS)_2_Ni (HD)	7.2 ± 0.3 ^d^	1.65 ± 0.05	3.68 ± 0.01	96
(TNS)_2_Ni (LD)	26.4 ± 0.2 ^d^	2.00 ± 0.05	3.05 ± 0.01	95

All values are expressed as mean ± standard error mean. Means with different superscripts are significantly different. The mean difference is significant at the *p* < 0.05 level. HD: High dose: 60 mg/kg, LD: Low dose: 30 mg/kg.

#### 2.2.3. Histological Examination

**Figure 2:**
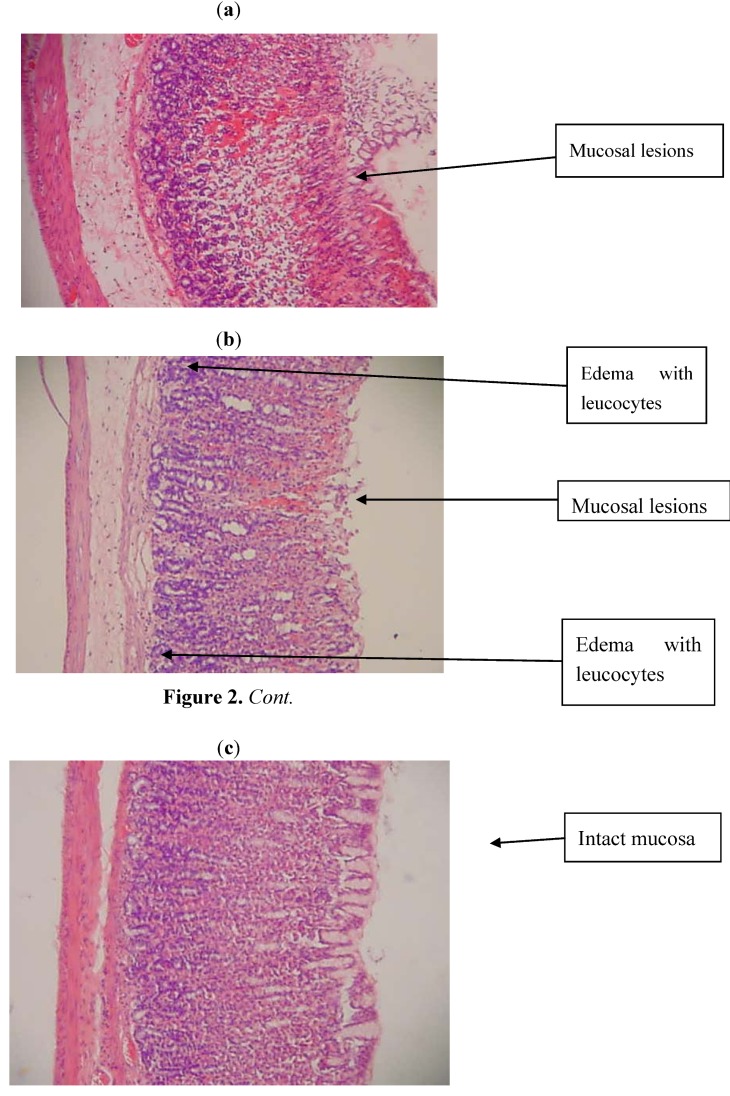
(**a**) Histological section of gastric mucosa in a rat pre-treated with 10 ml/kg of 10% Tween-20 (ulcer control) only. There is severe disruption to the surface epithelium, and edema of the submucosal layer with leucocyte infiltration. (**b**) Histological section of gastric mucosa in a rat pre-treated with 10 ml/kg of Cimetidine (50 mg/kg). There is mild disruption to the surface epithelium with mild edema and leucocytes infiltration of the submucosal layer. (**c**) Histological section of gastric mucosa in a rat pre-treated with 10 ml/kg of TNS (60 mg/kg). There is no disruption to the surface epithelium with no edema and no leucocytes infiltration of the submucosal layer.

Rats pre-treated with 10% Tween-20 prior to administration of absolute ethanol showed severe mucosal damage and the submucosa was markedly thickened by edema (Figure 2a). Deep hemorrhagic lesions in the mucosal layer and infiltration of leucocytes were observed in the submucosal layer. As seen in Figure 2c, rats pre-treated with TNS had milder mucosal damage, and less submucosal edema and leucocyte infiltration.

### 2.3. Discussion

The results of this study showed that TNS ligand is more effective as an anti-ulcer drug than its nickel (II) complex, yet, the ligand and its complex can be considered as potential anti-ulcer drugs compared to cimetidine as shown in Table 1.

This can be explained in the bases of reducing the acidity of the stomach juice as it can be implied from the pH values. These results also confirm the antisecretory ability of these compounds as reflected from the significant reduction of gastric juice acidity, beside the enhancement of mucus secretion. Ethanol is commonly used for inducing ulcers in experimental rats and leads to intense gastric mucosal damage. Ethanol shows its harmful effects either through direct generation of reactive metabolites, including free radical species that react with most of the cell components, changing their structures and functions, or promote enhanced oxidative damage [14,15]. It has been reported that the gastric cytoprotection might be mediated by at least two different mechanisms: one of them through prostaglandin synthesis, and the second one by increasing the production of mucosal glycoproteins [16,17]. Metal complexes were shown to enhance the formation of prostaglandine E_2_ and are likely to participate in gastroprotective activity [18,19]. The results of the current study showed that rats pre-treated with TNS had significantly reduced gastric mucosal injury with less leucocyte infiltration of the submucosal layer. Cytoprotection was assessed by reduction in the macroscopically and microscopically visible lesions. Absolute alcohol would extensively damage the gastric mucosa leading to increased neutrophil infiltration into the gastric mucosa. Neutrophils are a major source of inflammatory mediators and can release potent reactive oxygen species such as superoxide, hydrogen peroxide and myeloperoxidase derived oxidants. These reactive oxygen species are highly cytotoxic and can induce tissue damage [20]. Furthermore, neutrophil accumulation in gastric mucosa has been shown to induce microcirculatory abnormalities [21]. Suppression of neutrophil infiltration during inflammation was found to enhance gastric ulcer healing [22]. Further studies are required to explore the detailed mechanism of action responsible for the anti-ulcer activity of these compounds.

## 3. Experimental

### 3.1. Materials and Methods

Cimetidine was used as the reference anti-ulcer drug, which belongs to a class of medication called histamine H_2_-receptors [23] and was obtained from the University Malaya Medical Centre (UMMC) Pharmacy. The drug was dissolved in 10% Tween-20 and administered orally to the rats at a dosage of 50 mg/kg body weight (5 mL/kg). Tryptamine and 5-nitrosalicylaldehyde were purchased from Sigma-Aldrich (Kuala Lumpur, Malaysia) and used without further purification.

### 3.2. Preparation of the Ligand and Its Nickel Complex

The ligand and its nickel (II) complex were prepared according to the method described in the literature [13]. Briefly: a solution of tryptamine (0.30 g, 1.9 mmol) in acidified ethanol (50 mL, pH 4.5) was added to a boiling solution of 5-nitrosalisylaldehyde (0.32g, 1.9 mmol) in acidified ethanol (50 mL, pH 4.5). The mixture was refluxed for 2 hours. The solid product that had formed was filtered off, and recrystallized from THF (yield 54%). It is soluble in acetone, THF and DMSO, but not soluble in either ethanol or water. *Anal.* Calc. for C_17_H_15_N_3_O_3_: C, 66.01; H, 4.89; N, 13.58%. Found: C, 65.97; H, 4.79; N, 13.63%. The complex was prepared as follows: to solution of nickel acetate tetrahydrate was added a solution of the Schiff base TNS in THF in 1:2 metal to ligand ratio. A few drops of triethylamine were then added. The mixture was refluxed for 5 hours. The product that had formed was filtered off, washed with THF and dried over anhydrous silica gel. Yield 76%. The complex is insoluble in most organic solvents, but soluble in DMSO and DMF. *Anal.* Calc. for C_34_H_28_N_6_O_6_Ni: C, 60.41; H, 4.14; N, 12.43%. Found: C, 60.32; H, 4.24; N, 12.21%. After separation and purification, both TNS and its nickel (II) complex were suspended in 10% Tween-20 solution. They were then orally administered to the rats at dosage of low dose (30 mg/kg) and high dose (60 mg/kg) body weight.

### 3.3. Experimental Animals

Adult male Sprague-Dawley rats were obtained from the Animal House, Faculty of Medicine, University of Malaya, Kuala Lumpur (Ethics No. PM 28/9/2007 MAA (R)). The rats weighed between 200–220 g. They were fasted for 24 hours before the experiment [24], but were allowed free access to drinking water till two hours before the experiment. During the fasting period, the rats were placed in six cages (groups), with wide-mesh wire bottoms to prevent coprophagia. Each cage contains six randomly selected rats.

Gastric ulcer was induced according to the method described by Robert *et al.* [25] with some modification in adult male Sprague-Dawley rats. Group 1 rats were ulcer control that received 10 mL/kg of 10% Tween-20 orally by orogastric intubations, whereas Group 2 rats received oral doses of 50 mg/kg cimetidine (10 mL/kg) as positive control. Group 3 and 4 rats received oral high dose (60 mg/kg) and low dose (30 mg/kg) of TNS respectively. Group 5 and 6 received oral high dose (60 mg/kg) and low dose (30 mg/kg) of the nickel (II) complex, respectively. One hour after this pre-treatment, rats were gavaged with absolute ethanol (5 mL/kg) in order to induce gastric ulcers. The rats were euthanized 60 minutes later [26] by overdoses of diethyl ether and their stomachs were immediately excised. Each stomach was opened along the greater curvature, washed with distilled water and fixed in 10% buffered formalin for 15 minutes.

The stomachs were removed and the gastric juice was obtained from each stomach. The surface area (mm^2^) covered by each lesion was measured [27], and the sum of erosion areas per rat stomach was estimated by using a microscope at magnification × 1.8. Percentages of ulcerated surface (US) were calculated as: US (mm^2^) = (total area covered by ulcers/total corpus mucosal surface) × 100. The ulcer index (UI) for each animal was then calculated as the mean ulcer score. Percentage inhibition (%I) was determined as [(UI in control – UI in test group)/UI in control group] × 100 [28].

After the stomach contents were collected, they were centrifuged and gastric juice was separated from the mucus. The mucus content was weighed and expressed in terms of grams [29] and the pH of the stomach juice was recorded.

### 3.4. Acute Toxicity Test

The acute toxicity was estimated according to the reported guidelines for testing of chemicals acute oral toxicity [30,31]. The test was used to determine a safe dose for the compounds TNS and its nickel (II) complex. Thirty six ICR mice (18 males and 18 females) were assigned equally into three groups (6 mice in each group) labeled as vehicle 10% Tween-20 (5 mL/kg); 2 g/kg and 5 g/kg for each of the compounds in 10% Tween-20 preparation. These animals were subjected to overnight fasting (food but not water) prior to dosing. Food was withheld for a further 3 to 4 hours after dosing. The animals were observed for 30 minutes and 2, 4, 24 and 48 h after the administration for the onset of clinical or toxicological symptoms. Mortality, if any was observed over a period of 2 weeks.

### 3.5. Histological Evaluation of Gastric Lesions

Specimens of the gastric walls from each rat were fixed in 10% buffered formalin and processed in a paraffin tissue processing machine. Sections of the stomach were made at a thickness of 5 µm and stained with hematoxylin and eosin for histological evaluation [23]. 

### 3.6. Ethics

Throughout the experiments, all animals received human care according to the criteria outlined in the “Guide for the Care and Use of laboratory Animals” prepared by the National Academy of Sciences and published by the U.S. National Institutes of Health.

### 3.7. Statistical Analysis

All values were reported as mean ± S.E.M. The statistical significance of differences between groups was assessed using one-way ANOVA. A value of *p <* 0.05 was considered significant.

## 4. Conclusions

Novel Schiff base derived from tryptamine and 5-nitrosalicylaldehyde and its nickel (II) complex have been synthesized and studied for their gastroprotective activity. The compounds were significantly protecting the gastric mucosa against ethanol-induced injury in comparison to cimetidine. Such protection was ascertained by the reduction of gastric ulcer areas and by the production of mucus. Acute toxicity studies reveal that the compounds were found to be safe and no behavioral observations were detected for toxicity signs.
